# The impact of metformin use on the outcomes of locally advanced breast cancer patients receiving neoadjuvant chemotherapy: an open-labelled randomized controlled trial

**DOI:** 10.1038/s41598-022-11138-3

**Published:** 2022-05-10

**Authors:** Hadeer Ehab Barakat, Raghda R. S. Hussein, Ahmed Abdullah Elberry, Mamdouh Ahmed Zaki, Mamdouh Elsherbiny Ramadan

**Affiliations:** 1 Department of Clinical Pharmacy, Faculty of Pharmacy, Ahram Canadian University, Giza, Egypt; 2 Department of Clinical Pharmacy, Faculty of Pharmacy, Beni-Suef University, Beni-Suef, Egypt; 3 Department of Clinical Pharmacy, Faculty of Pharmacy, Modern University for Technology and Information, Cairo, Egypt; 4 Department of Pharmacy Practice, Batterjee Medical College, Pharmacy Program, Jeddah, Saudi Arabia; 5 Department of Clinical Pharmacology, Faculty of Medicine, Beni-Suef University, Beni-Suef, Egypt; 6 Ahram Canadian University, Giza, Egypt; 7 Department of Medical Oncology, Faculty of Medicine, Beni-Suef University, Beni-Suef, Egypt

**Keywords:** Breast cancer, Health care

## Abstract

Recently, several clinical trials have attempted to find evidence that supports the anticancer use of metformin in breast cancer (BC) patients. The current study evaluates the anticancer activity of metformin in addition to neoadjuvant chemotherapy (NACT) in locally advanced BC patients. Additionally, we assess the safety and tolerability of this combination and its effect on the quality of life (QoL) of BC patients. Eighty non-diabetic female patients with proven locally advanced BC were randomized into two arms. The first arm received anthracycline/taxane-based NACT plus metformin. The second arm received anthracycline/taxane-based NACT only. Overall response rate (ORR), clinical complete response (cCr), pathological complete response (pCR), and breast conservative rate (BCR) were evaluated between both groups, and correlated with serum metformin concentration. ORR, cCr, pCR, and BCR increased non-significantly in the metformin group compared to the control group; 80.6% vs 68.4%, 27.8% vs 10.5%, 22.2% vs 10.5%, and 19.4% vs 13.2%, respectively. A trend towards cCR and pCR was associated with higher serum metformin concentrations. Metformin decreased the incidence of peripheral neuropathy, bone pain, and arthralgia, although worsened the gastrointestinal adverse events. Metformin combination with NACT has no effect on the QoL of BC patients. Metformin combination with NACT is safe, tolerable, and improves non-significantly the clinical and pathological tumor response of BC patients.

## Introduction

Repurposing existing medications that may have antineoplastic properties motivates recent clinical research directions to find safe and effective auxiliary anticancer treatments. Oral biguanide metformin, the first-line treatment for diabetes mellitus type 2, exhibits several appealing antineoplastic attributes that make it challenging for repurposing as an anticancer treatment^1,2^.

In the literature, several retrospective clinical studies have discussed with great attention the anticancer effect of metformin in breast cancer (BC)^3–5^. The first clinical evidence of the anticancer effect of metformin, Jiralerspong et al. (2009) retrospective trial, recommended the addition of metformin to neoadjuvant chemotherapy (NACT) as it enhances significantly the pathological complete response (pCR) for diabetic BC patients^5^. This study paved the way for further prospective trials to determine the anticancer activity of metformin according to the patients’ characteristics and BC subtypes^6–9^.

Most of these trials were conducted in the adjuvant and metastatic BC settings. Although the neoadjuvant approach is recommended to test the activity of new drugs, as it can augment the capacity to verify the beneficial combinations of drugs in thoroughly designed early BC clinical trials^10,11^. Therefore, we can avoid the overestimation of the potential anticancer effect of metformin, and specify BC groups more likely to benefit from metformin addition to their anticancer regimen^12^.

In such regards, the METTEN prospective study was conducted on human epidermal growth factor receptor 2 positive (HER2 +) non-diabetic BC patients receiving trastuzumab and neoadjuvant chemotherapy in combination with metformin^12^. This study observed that pCR was higher numerically in metformin users compared to nonusers. However, the study was underpowered, so it could not conclude metformin effectiveness. Additionally, the METEOR study, which considered neoadjuvant metformin combination with letrozole for postmenopausal estrogen receptor-positive (ER +) breast cancer patients, observed that the overall clinical response rate was higher numerically but not significantly in the metformin group compared to the placebo group^13^.

From another perspective, potential mechanistic elucidations of metformin's anticancer activity include its ability to overcome resistance to certain systemic anticancer treatments or to synergistically improve their anticancer activity^3,14,15^. Therefore, it is of crucial importance to not only study the characteristics of the patients and tumor, but also to identify the anticancer treatments that could achieve a safe and effective response with metformin combination. Hirsch et al. (2009) preclinical study demonstrated that the combination therapy of metformin and doxorubicin reduced the tumor mass and prevented the disease recurrence much more effectively than either drug alone in a BC xenograft mouse model^14^. Moreover, Rocha et al. (2011) deduced the presence of the synergistic effect of metformin combination with paclitaxel on adenosine monophosphate-activated protein kinase (AMPK) signaling that leads to increasing the downregulation of the mammalian target of rapamycin (mTOR) pathway with the combination treatment rather than either drug alone^15^. Moreover, Kalinisky et al. (2014) raised an important question of whether ethnicity can affect the impact of metformin on BC biology^16^. Racial and ethnic differences have been observed in the response to metformin in diabetes treatment^17^. Therefore, the main objective of this study is to determine whether the combination of metformin with anthracycline/taxane-based neoadjuvant chemotherapy in Egyptian breast cancer patients could enhance the tumor response.

On the other hand, health-related quality of life (HRQoL) has been considered a main clinical outcome of cancer research, reflecting patient reported outcomes (PROs)^18^. The vital importance of PROs is the expression of patient satisfaction and endurance to the disease or treatment impacts on the patients’ daily life.

Therefore, in the current study, the primary aim is to determine the effect of adding metformin to the anthracycline/taxane-based neoadjuvant chemotherapy on the clinical benefit in terms of overall response, clinical complete response, and pathological complete response in locally advanced, operable, Egyptian breast cancer patients. The secondary aim is to determine the breast conservative rate (BCR) between metformin users and nonusers and to evaluate the safety profile and tolerability of adding metformin to the neoadjuvant chemotherapeutic regimen. Furthermore, the quality of life of breast cancer patients is compared between metformin users and nonusers using the European Organization for Research and Treatment of Cancer quality of life questionnaire (EORTC QLQ) core module EORTC QLQ-C30 and the breast cancer-specific module EORTC QLQ-BR23 module.

## Patients and methods

### Trial design and participants

A prospective, single-center, open-labelled, block-randomized, controlled study was conducted from June 2019 till November 2020 in Beni-Suef university hospital, Beni-Suef, Egypt. Eighty non-diabetic female patients of age between 18 and 65 years, with proven locally advanced breast cancer, were enrolled in the study. The metformin arm received the first-line NACT regimen (4 cycles doxorubicin 60 mg/m^2^/ cyclophosphamide 600 mg/m^2^, followed by 12 cycles of weekly paclitaxel 80 mg/m^2^) plus metformin (1000 mg twice daily)^7,9^. The control arm received the NACT regimen only (4 cycles doxorubicin 60 mg/m^2^/ cyclophosphamide 600 mg/m^2^, followed by 12 cycles of weekly paclitaxel 80 mg/m^2^). Escalation of metformin dose was done as follows; 500 mg twice daily in the first week, followed by 500 mg three times daily in the second week, followed by 1000 mg twice daily till the last chemotherapy cycle. Then, all the patients were eligible for breast surgery either mastectomy or lumpectomy after 3–4 weeks post last chemotherapy cycle. Adjuvant trastuzumab was received by HER2 positive patients in our study. The study was approved prospectively by the Institutional Review Board (IRB) of the Faculty of Pharmacy, Beni-Suef University (REC-H-PhBSU-20012), and patient consent was obtained from all patients enrolled in the current study. The study was conducted in accordance with the Declaration of Helsinki, Good Clinical Practice norms, and local and national regulatory requirements. The study was registered in Clinicaltrials.gov, 22/09/2020, NCT04559308. Approval of the written informed consent was acquired by all the patients before enrollment to the study.

### Eligibility criteria and patients selection

Patients who met the following criteria were eligible for the study: previously untreated, operable, proven locally advanced BC, with palpable and clinically measurable tumors. Locally advanced breast cancers are those with stage IIB (T3N0) and stage IIIA though IIIC^19^. Patients were excluded from this study if the patients were diabetic (hemoglobin A1c (HbA1c) ≥ 6.5% or fasting plasma glucose ≥ 126 mg/dL), early-stage, or metastatic breast cancer. Additionally, exclusion includes patients with renal impairment (estimated glomerular filtration rate (eGFR) < 45 mL/min/1.73 m^2^), hepatic impairment (alanine aminotransferase/aspartate aminotransferase **(**ALT/AST) > 1.5 × ULN), impaired cardiac function (left ventricular ejection fraction (LVEF) < 55%), hypersensitivity to metformin, or at risk of lactic acidosis.

### Randomization

Patients were randomly assigned into 1:1 ratio to either the metformin arm or the control arm with a randomized block design. The stratification factors of our study were menopausal state (pre-menopausal or post-menopausal); body mass index (normal weight, overweight, or obese); and HER2 state (positive or negative), to decrease the imbalance between treatment groups for factors that might influence both the prognosis or treatment responsiveness with the addition of metformin to the NACT. Pre-menopausal was defined as a woman during the reproductive period before the menopause^20^; post-menopausal was defined as 12 months of spontaneous amenorrhea or 6 months of spontaneous amenorrhea with serum FSH levels > 40 mIU/ml or 6 weeks postsurgical bilateral oophorectomy with or without hysterectomy^20,21^. Body mass index (BMI) was categorized as normal weight, ranges from 18.5 kg/m2 to < 25 kg/m2; overweight, ranges from 25.0 kg/m2 to < 30 kg/m2; and obese are ≥ 30 kg/m2^22^. HER2 positive was defined as breast tumors with HER2 overexpressed or amplified.

**Figure 1:**
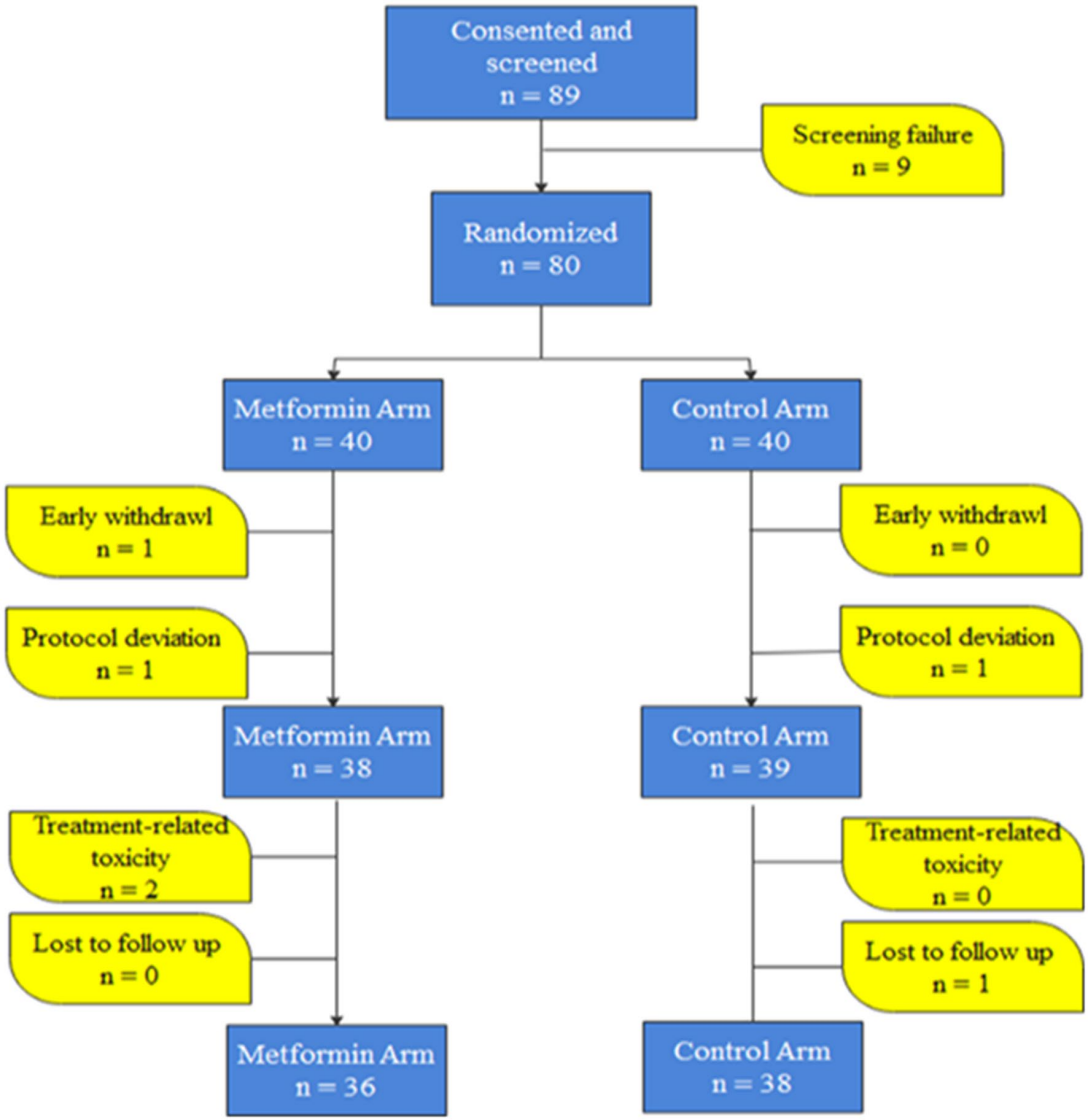
The CONSORT diagram illustrating the organization of the patients in the study.

Accordingly, a total of twelve combined strata were identified. Hence, ninety-six randomization codes were generated with a block size of 8 patients (4 per arm) for each combined strata. The CONSORT diagram shown in Fig. 1 illustrates the disposition of patients in the study. Eighty-nine patients were consented and screened. Nine patients did not meet the inclusion criteria and were excluded from randomization. Furthermore, six patients were excluded from randomization because of protocol deviation, early withdrawal, treatment-related toxicity, or lost to follow up. Therefore, seventy-four patients were enrolled and accomplished the study, in which thirty six of them were allocated in the metformin arm and thirty eight in the control arm.

### Objective and outcome evaluation

The primary aim of this study is to evaluate the clinical benefit and pathological tumor response of combining metformin and neoadjuvant chemotherapy treatment of non-diabetic locally advanced breast cancer patients. The primary breast lesion and the axillary lymph nodes were assessed by physical examination, diagnostic bilateral mammography with ultrasound (sonomamography) prior to the first chemotherapy cycle. The size of the breast lump diameter was recorded in two dimensions with palpation and calipers by an experienced clinician. The tumor size was measured by two-dimensional mammography and three-dimensional sonography. The sum of the diameters (longest for non-nodal lesions, short axis for nodal lesions) for all target lesions was measured and reported as the baseline sum diameters. The clinical response rate during the 24 weeks was determined by measuring the changes in the sum of diameters of target lesions from baseline. Physical examination, with calipers and palpation, was done prior to each chemotherapy cycle every 3 weeks. In addition, the assessment of the tumor size was done by ultrasound and mammography at week 12 and at week 24 (prior to the surgical decision).

The clinical response was categorized into four categories according to the RECIST v1.1 criteria; complete response (CR), partial response (PR), stable disease (SD), and progressive disease (PD)^23^. With reference to the baseline tumor size, the disappearance of all target lesions, and lymph nodes size < 10 mm short axis indicates CR; the decrease in the sum of diameters of target lesions at least 30% indicates PR; at least 20% increase in the sum of diameters of target lesions indicates PD; and tumor response with neither sufficient shrinkage to qualify for PR nor sufficient increase to qualify for PD indicates SD. The overall response rate (ORR) is defined as “the proportion of patients who have achieved a partial or complete response to therapy”. ORR is a direct measure of anticancer agent tumoricidal activity^24^. On the other side, the pathological complete response (pCR) is defined as “the absence of invasive cancer at the primary site and in the axilla following the completion of neoadjuvant systemic chemotherapy”. pCR definition includes also the presence of residual ductal carcinoma in situ according to the American Joint Committee on Cancer (AJCC), eighth edition^25^. All adverse events (AEs) were recorded and graded using the National Cancer Institute common terminology criteria for adverse events (NCI-CTCAE) version 4.0.

The diagnosis of locally advanced BC was done by core-needle biopsy. Immunohistochemical (IHC) staining was used to determine the expression status of estrogen receptor (ER), progesterone receptor (PgR), and HER2 status. Breast tumors with 3 + HER2 scores were considered positive, while 1 + or 0 were considered negative. In tumors with 2 + scores, HER2 amplification was done by fluorescence in situ hybridization. The evaluation of the ER, PgR, and HER2 status expressions were performed using the American Society of Clinical Oncology/College of American Pathologists (ASCO/CAP) guidelines^26,27^. Intrinsic subtypes of breast cancer were determined according to surrogate clinico-pathological definitions adapted from the 2013 St Gallen Consensus Conference^28,29^. Pretreatment diagnostic biopsies were reviewed as regards the histological type of the tumor according to World Health Organization (WHO) classification of the tumors of the breast^30^, and grading which was evaluated according to Nottingham combined histologic grade^31^. Clinical staging was defined according to AJCC manual, eighth edition^25^.

### Analytical determination of circulating metformin in serum

Cancer patients receiving the same dosage could experience interpatient pharmacokinetic variability of plasma concentration^32,33^. Therefore, a correlation of the tumor response with the serum concentration of metformin was done. At the end of the chemotherapeutic intervention (24 weeks), blood samples were collected 2 to 4 h post last metformin dose administration. This time is correlated with the maximum concentration (Cmax) of metformin (Tmax = 1.75 h to 3.5 h)^34^. Serum samples were stored at –20° C until being assayed. Assay of serum metformin concentrations was accomplished using high-performance liquid chromatography (HPLC) with ultra-violet (UV) detector^35^. The chromatographic separation was conducted on a reversed-phase phenyl column at 40^◦^C. The preparation of samples was complemented through protein precipitation with acetonitrile. The mobile phase consisted of a mixture of phosphate buffer 0.02 M (pH 7.0) and acetonitrile (50:50, v/v) at a flow rate of 1.0 ml/min. It was prepared daily and degassed before use. Wavelength was set at 236 nm^35^.

### Patient-Reported Outcomes (PROs) assessment using QLQ-C30 and QLQ-BR23

A secondary objective of this study is the assessment of the PROs to evaluate the effect of the addition of metformin to the neoadjuvant chemotherapy regimen on the quality of life (QoL) of BC patients. The PROs were assessed using the EORTC QLQ-C30 v 3.0^36^ and the EORTC QLQ-BR23 BC module^37^. The EORTC QLQ-C30 is a 30-item questionnaire consists of a global QoL scale, 5 functional scales (physical, role, emotional, cognitive, and social functioning), 3 multi-item symptom scales (fatigue, nausea/vomiting, and pain), and 6 single-item symptom scales (dyspnea, sleep disturbance, appetite loss, constipation, diarrhea, and financial difficulties). On the other side, the EORTC QLQ-BR23 questionnaire is composed of 23-items related to BC or its treatment. The QLQ-BR23 module consists of 4 functional scales (body image, sexual functioning, sexual enjoyment, and future perspective), 3 multi-items symptoms scales (systemic side-effects, breast symptoms, and arm symptoms), and one single-item symptom scale (upset by hair loss).

In the PROs assessment step, all the patients were interviewed twice; the first interview was done at baseline, on the day of the first chemotherapy cycle and the second interview was done on the day of the last chemotherapy cycle. Then, scoring of the EORTC QLQ-C30 and EORTC QLQ-BR23 were performed according to the EORTC scoring manual^33^. After the scoring procedures, all the responses to Likert scale items were linearly transformed to a 0–100 scale. Higher scores for functional scales and the global scale indicate better QoL. On the other side, higher scores for symptom scales and single items indicate higher severity of the symptoms and worse QoL.

### Statistical analysis

Baseline patient demographics and tumor characteristics were analyzed with frequencies and percentages. For categorical data, a comparison between metformin users and nonusers in the baseline characteristics was done using the Chi-Squared test or Fisher’s exact test according to the appropriateness. Normality evaluation for continuous variables was performed using the Kolmogorov–Smirnov test. Continuous data were represented as mean ± standard deviation (SD) using unpaired t-test or as median using Mann–Whitney U test when the data were not normally distributed. Univariate and multivariate binary logistic regressions were performed to predict the effect of metformin addition to NACT on the tumor response, as well as to evaluate the effects of different baseline characteristics on the clinical and pathological outcomes.

The sample size was calculated based on the expected pCR improvement to detect a change in the binary response variable from the baseline value of 0.10 to 0.35, with a statistical power (1 − beta) of 80% assuming a two-sided confidence level (1 − alpha) of 95% with a drop-out rate of 10%. Therefore, the sample size was calculated as 80 patients (of which 50% were in the metformin group and 50% were in the control group).

In the literature, the frequency of pCR shows various percentages according to several factors. For example, pCR is low in patients with low-grade HR + tumors, and could be doubled in high-grade HR + subgroup; the pCR percentage showed 7.5% with low-grade HR + /HER2-; 18.3% with HR + /HER2 + ; 30% with HR-/HER2 + ; and 33% with triple negative breast cancer (TNBC)^38^. Besides, approximately up to 75% of the breast tumors are ER + ^39^. In the current trial, a pCR of 10% was set as the baseline value in the control group, based on several studies that showed a pCR of 7.1%, 8.3%, 10.1%, 11%, and 12.4% in locally advanced BC patients^40–44^. On the other hand, the setting in this study considered a 25% improvement in the pCR of the metformin group was based on several studies. The study by Jiralerspong et al. (2009), showed a pCR of 24% in diabetic BC patients receiving metformin compared to 8% in the control arm^5^. In addition, the trial by Liubota et al. (2016), found that the rate of pCR in BC patients with metabolic syndrome receiving neoadjuvant systemic treatment was 31% in the metformin group versus 6% in the control group^45^. Furthermore, The trial by Laat et al. (2016), found a statistically significant association between the pCR and metformin, pCR was achieved in 64.3% in the metformin arm versus 23.1% in the control arm^46^.

Analysis of covariance (ANCOVA) test was used to compare the mean scores of the PROs among the QoL items of the two groups of patients (metformin receiving group and the control group). The analysis was done by considering the mean scores at the last cycle as the dependent variable, the group of the patient as the independent variable, and the baseline mean scores as the covariate. P-value (≤ 0.05) is considered statistically significant with a confidence interval of 95%. All the statistical analyses were carried out using SPSS (IBM Corp. released 2016. IBM SPSS Statistics for Windows, Version 26.0).

## Results

Eighty patients with locally advanced breast cancer were included in the current study. Six patients were excluded due to early withdrawal (n = 1), protocol deviation (n = 2), treatment related toxicity (n = 2), or lost to follow up (n = 1). Seventy-four patients were randomized, 36 patients in the metformin arm and 38 patients in the control arm were evaluated for the primary and secondary outcomes of the study as shown in Fig. 1.

### Baseline patient demographics and tumor characteristics

Table 1 shows a comparison between the metformin users and the nonusers in terms of their demographic data and tumor characteristics at baseline. There is no significant difference in the baseline characteristics between both groups.

**Table 1:** Baseline patient demographic and tumor characteristics by study group.

Variable	Metformin group (N = 36)	Control group (N = 38)	*P*-value
**Age**			0.363
≥ 50	18 (50.00%)	15 (39.47%)	
< 50	18 (50.00%)	23 (60.53%)	
Mean ± S.D	49.14 ± 11.22	47.13 ± 10.53	0.426
**Ki67**			0.222
< 20%	14 (38.89%)	21 (55.26%)	
≥ 20%	22 (61.11%)	17 (44.74%)	
Mean ± S.D	26.66 ± 13.88	25.95 ± 19.86	0.691
**BMI Category**			0.982
Normal weight	8 (22.2%)	8 (21.05%)	
Overweight	13 (36.11%)	13 (34.21%)	
Obese	15 (41.67%)	17 (44.73%)	
Mean ± S.D	30.44 ± 5.52	30.26 ± 5.87	0.846
**Menopausal state**			0.65
Premenopause	18 (50%)	20 (52.63%)	
Postmenopause	18 (50%)	18 (47.37%)	
**Hormone receptor status**			0.124
ER and/or PgR positive	33 (91.67%)	30 (78.95%)	
ER and PgR negative	3 (8.33%)	8 (21.05%)	
**HER2 status**			0.778
Positive	14 (38.89%)	16 (42.11%)	
Negative	22 (61.11%)	22 (57.89%)	
**Intrinsic subtype**			0.442
Luminal A	15 (41.67%)	15 (39.4%)	
Luminal B/HER2 negative	5 (13.89%)	3 (3.95%)	
Luminal B/HER2 positive	13 (36.11%)	12 (31.58%)	
HER2 positive/non-luminal	1 (2.77%)	4 (10.53%)	
TNBC	2 (5.56%)	4 (10.53%)	
**Clinical tumor stage**			0.549
T2	2 (5.56%)	4 (5.26%)	
T3	20 (55.56%)	17 (44.74%)	
T4	14 (38.89%)	17 (44.74%)	
**Clinical nodal stage**			0.386
N0	6 (16.67%)	5 (13.16%)	
N1	22 (61.11%)	22 (57.89%)	
N2	8 (22.22%)	8 (21.05%)	
N3	0 (0%)	3 (7.89%)	
**Clinical prognostic stage**			0.338
IIB	5 (13.89%)	5 (13.16%)	
IIIA	16 (44.44%)	15 (39.47%)	
IIIB	15 (41.67%)	18 (47.37%)	
**Histological grade**			0.093
Grade 1 and 2	22 (61.11%)	30 (78.95%)	
Grade 3	14 (38.89%)	8 (21.05%)	

*Ki67* proliferation index, *BMI* body mass index, *ER* estrogen receptor, *PgR* progesterone receptor, *HER2* human epidermal growth factor receptor 2, *TNBC* triple negative breast cancer, *SD* standard deviation.

### Pathological complete response (pCR) evaluation

As shown in Table 2, although metformin shows a numerically higher pCR post neoadjuvant treatment (n = 8, 22.2%) compared to the control group (n = 4, 10.5%), it did not reach the level of significance (OR 2.429, 95% CI 0.662—8.914, p = 0.181). On the other side, HER2 + state was a significant predictor of the pCR (OR 3.636, 95% CI 1.022—13.452, p = 0.044). In addition, high histological grade tumors increased significantly the pCR compared to lower histologic grades (OR 6.857, 95% CI 1.796—26.182, p = 0.005). Furthermore, a higher baseline proliferation index (Ki67) was associated with a significant increase of the pCR (OR 8.77, 95% CI 1.018—76.923, p = 0.05). According to the multivariate analysis shown in Table 3, it was observed that metformin addition to NACT, in association with the predictors of pCR, remains non-significant. Besides, the multivariate model illustrates that only a high baseline histological grade was independently predictive of pCR after adjusting other predictors (OR 6.9, 95% CI 1.323 -36.035, p = 0.022).

**Table 2:** Univariate analysis of variables associated with response according to the different endpoints.

Variable	Pathologic Complete Response (pCR)						Overall Response Rate (ORR)					Clinical Complete Response (cCR)				
	pCR N (%)	Non- pCR N (%)	OR	95% CI		*P*-value	ORR N (%)	Non-ORR N (%)	OR	95% CI	*P*-value	cCR N (%)	Non-cCR N (%)	OR	95% CI	*P*-value
**Group**																
Metformin	8 (22.2%)	28 (77.8%)	2.429	0.662 to 8.914		0.181	29 (80.6%)	7 (19.4%)	1.912	0.655 to 5.585	0.236	10 (27.8%)	26 (72.2%)	3.269	0.921 to 11.606	0.058
Control	4 (10.5%)	34 (89.5%)	1				26 (68.4%)	12 (31.6%)	1			4 (10.5%)	34 (89.5%)	1		
**Age**																
< 50	7 (17.1%)	34 (82.9%)	1.153	0.330 to 4.032		0.824	28 (68.3%)	13 (31.7%)	0.479	0.159 to 1.441	0.190	8 (19.5%)	33 (80.5%)	1.094	0.337 to 3.530	0.885
≥ 50	5 (15.2%)	28 (84.8%)	1				27 (81.8%)	6 (18.2%)	1			6 (18.2%)	27(81.8%)	1		
**BMI**																
Obese/overweight	9 (15.5%)	49 (84.5%)	0.796	0.188 to 3.368		0.756	43 (74.1%)	15 (25.9%)	0.956	0.267 to 3.421	0.944	10 (17.2%)	48 (82.7%)	0.625	0.167 to 2.342	0.486
Normal weight	3 (18.8%)	13 (81.2%)	1				12 (75%)	4 (25%)	1			4 (25.0%)	12 (75.0%)	1		
**Menopausal state**																
Postmenopause	5 (13.9%)	31 (86.1%)	0.714	0.204 to 2.496		0.598	29 (80.5%)	7 (19.5%)	1.912	0.655 to 5.585	0.236	6 (16.7%)	30 (83.3%)	1.333	0.412 to 4.310	0.631
Premenopause	7 (18.4%)	31 (81.6%)	1				26 (68.4%)	12 (31.6%)	1			8 (21.1%)	30 (78.9%)	1		
**ER**																
Positive	7 (12.3%)	50 (87.7%)	0.336	0.091 to 1.244		0.103	43 (75.4%)	14 (24.6%)	1.28	0.384 to 4.27	0.688	8 (14.0%)	49 (86.0%)	0.299	0.086 to 1.039	0.057
Negative	5 (29.4%)	12 (70.6%)	1				12 (70.6%)	5 (29.4%)	1			6 (35.3%)	11 (64.7%)	1		
**PgR**																
Positive	9 (17.6%)	42 (82.4%)	1.429	0.348 to 5.857		0.620	40 (78.4%)	11 (21.6%)	1.939	0.654 to 5.751	0.232	9 (17.6%)	42 (82.4%)	1.296	0.381 to 4.412	0.678
Negative	3 (13.0%)	20 (87.0%)	1				15 (65.2%)	8 (34.8%)	1			5 (21.7%)	18 (78.2%)	1		
**HER2**																
Positive	8 (26.7%)*	22 (73.3%)	3.636	1.022 to 13.452		0.044	21 (70.0%)	9 (30.0%)	0.686	0.24 to 1.965	0.483	8 (26.7%)	22 (73.3%)	2.303	0.707 to 7.507	0.166
Negative	4 (9.1%)	40 (90.9%)	1				34 (77.2%)	10 (22.8%)	1			6 (13.6%)	38 (86.4%)	1		
**Histological grade**																
Grade 3	8 (36.4%)**	14 (63.6%)	6.857	1.796 to 26.182		0.005	13 (59.1%)	9 (40.9%)	0.344	0.115 to 1.028	0.056	6 (27.3%)	16 (72.7%)	2.062	0.619 to 6.870	0.238
Grade 1 and 2	4 (7.7%)	48 (92.3%)	1				42 (80.7%)	10 (19.3%)	1			8 (15.4%)	44 (84.6%)	1		
**Clinical tumor stage**																
T2	0 (0%)	6 (100.0%)	0			0.999	5 (83.3%)	1 (16.7%)	2.045	0.21 to 20.054	0.539	1 (16.7%)	5 (83.3%)	1.35	0.124 to 14.73	0.806
T3	8 (21.6%)	29 (78.4%)	1.862	0.503 to 6.899		0.352	28 (75.7%)	9 (23.3%)	1.273	0.432 to 3.746	0.662	9 (24.3%)	28 (75.6%)	2.17	0.597 to 7.891	0.240
T4	4 (12.9%)	27 (87.1%)	1				22 (71.0%)	9 (29.0%)	1			4 (12.9%)	27 (87.1%)	1		
**Clinical nodal stage**																
N0 and N1	11 (20.0%)	44 (80.0%)	4.5	0.541 to 37.464		0.164	44 (80.0%)	11 (20.0%)	2.909	0.944 to 8.962	0.063	13 (23.6%)	42 (76.3%)	5.571	0.677 to 45.843	0.071
N2 and N3	1 (5.3%)	18 (62.1%)	1				11 (57.9%)	8 (42.1%)	1			1 (5.3%)	18 (94.7%)	1		
**Clinical stage**																
Stage IIB	2 (20.0%)	8 (80.0%)	1.125	0.189 to 6.699		0.897	6 (60.0%)	4 (40.0%)	0.75	0.175 to 3.222	0.699	1 (10.0%)	9 (90.0%)	0.5	0.053 to 4.732	0.546
Stage IIIA	4 (12.9%)	27 (87.1%)	0.667	0.169 to 2.631		0.563	27 (87.1%)	4 (12.9%)	3.375	0.943 to 2.082	0.062	7 (22.6%)	24 (77.4%)	1.313	0.387 to 4.451	0.663
Stage IIIB	6 (18.2%)	27 (81.8%)	1				22 (66.7%)	11 (33.3%)	1			6 (18.2%)	27 (81.8%)	1		
**Ki67**																
≥ 20%	11 (28.2%)*	28 (71.8%)	8.77	1.018 to 76.923		0.05	27 (69.2%)	12 (30.8%)	0.441	0.11 to 1.764	0.247	13 (33.3%)*	26 (66.7%)	12.8	1.459 to 111.1	0.021
< 20%	1 (2.9%)	34 (97.1%)	1				28 (80.0%)	7 (20.0%)	1			1 (2.9%)	34 (97.1%)	1		

*OR* Odds Ratio, *BMI* body mass index, *ER* estrogen receptor, *PgR* progesterone receptor, *HER2* human epidermal growth factor receptor 2, *Ki67* proliferation index.

*Significant as compared to the control group at *P*-value ≤ 0.05, CI: 95% confidence interval.

** Significant as compared to the control group at *P*-value ≤ 0.01, CI: 95% confidence interval.

**Table 3:** Multivariate analysis of the factors associated significantly with pCR on the univariate level.

Variable	Group and HER2		Group and Ki67		Group and Histological grade		Group, HER2, Ki67, and Histological grade	
	OR (95% CI )	*P*-value	OR (95% CI )	*P*-value	OR (95% CI )	*P*-value	OR (95% CI )	*P*-value
**Group**								
Metformin	2.690 (0.700 to 10.335)	0.15	3.474 (0.570 to 21.17)	0.177	1.807 (0.452 to 7.224)	0.403	3.697 (0.445 to 30.690)	0.226
Control	1		1		1		1	
**HER2**								
Positive	3.917* (1.030 to 14.899)	0.045					2.035 (0.349 to 11.872)	0.239
Negative	1						1	
**Ki67**								
≥ 20%			7.634 (0.824 to 70.752)	0.074			4.497 (0.423 to 47.753)	0.117
< 20%			1				1	
**Histological grade**								
Grade 3					6.195 **(1.591 to 24.123)	0.009	6.905* (1.323 to 36.035)	0.022
Grade 1&2					1		1	

*OR* Odds Ratio, *HER2* human epidermal growth factor receptor 2, *Ki67* proliferation index.

*Significant as compared to the control group at *P* value ≤ 0.05, CI: 95% confidence interval.

** Significant as compared to the control group at *P* value ≤ 0.01, CI: 95% confidence interval.

### Clinical response evaluation

As shown in Table 2, there was a trend towards a significant increase in cCR of BC patients in the metformin arm (27.8%, n = 10) compared to the control arm (10.53%, n = 4), (OR 3.269, 95% CI 0.921–11.606, p = 0.058). Additionally, ORR increased numerically but not significantly in the metformin group (80.56%, n = 29) compared to the control group (68.42%, n = 26), (OR 1.912, 95% CI 0.655 to 5.585, p = 0.236). The proliferation index (Ki67) was the only predictor of improved cCR.

### Breast conservative rate (BCR) evaluation

**Figure 2:**
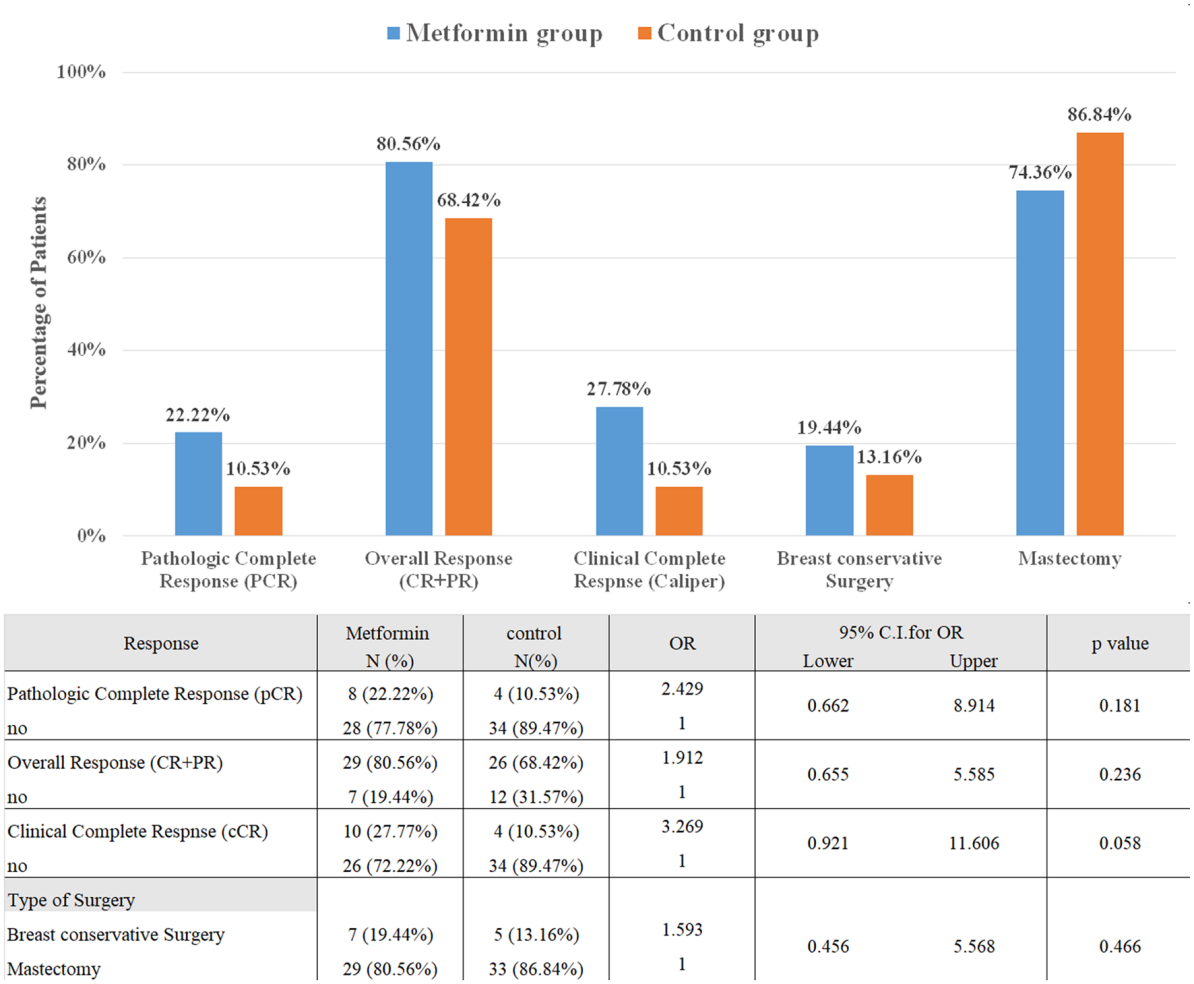
Effect of metformin on the different endpoints.

As shown in Fig. 2, 19.4% (n = 7) of patients in the metformin group performed breast conservative surgery compared to 13.15% (n = 5) in the control arm. No significant difference was observed between both groups in the BCR (OR 1.593, 95% CI 0.456—5.568, p = 0.466).

### Safety and tolerability

As shown in Table 4, the most common adverse events experienced by the metformin group were gastrointestinal tract (GIT) side effects, especially nausea, vomiting, and diarrhea. Interestingly, metformin improves some adverse events such as neuropathy, bone pain, arthritis, and myalgia. Chemotherapy-induced eripheral neuropathy (CIPN) decreased in the metformin group compared to the control group. In the metformin group, it was observed that 30.56% (n = 11) experienced grade I CIPN, and 13.89% (n = 5) experienced grade II CIPN. On the other side, 44.74% (n = 17) of patients in the control group experienced grade I CIPN, and 21.05% (n = 8) experienced grade II CIPN. In addition, 13.89% (n = 5) in the metformin group experienced grade I bone pain compared to 26.32% (n = 10) in the control group. Moreover, 11.11% (n = 4) experienced grade I arthralgia, and no patients experienced higher grade arthralgia in the metformin group. In the control group, 18.42% (n = 7) experienced grade I arthralgia, and one patient experienced grade II arthralgia. As well, Grade I myalgia was experienced by 13.89% (n = 5) in the metformin group compared to 18.42% (n = 7) in the control group. Grade II myalgia was experienced by 5.56% (n = 2) in the metformin group compared to 7.89% (n = 3) in the control group. In short, even though metformin addition to anthracycline/taxane-based chemotherapy showed worse GIT adverse events, an improvement of some low-grade (grade I and II) adverse events was also observed.

**Table 4:** Common adverse events reported in relation with the treatment group.

Adverse Event	Metformin group (N = 36)				Control group (N = 38)			
	Grade 1	Grade 2	Grade 3	Grade 4	Grade 1	Grade 2	Grade 3	Grade 4
Anemia	14 (38.89%)	8 (22.22%)	2 (5.56%)	0 (0.00%)	13 (34.21%)	10 (26.32%)	3 (7.89%)	0 (0.00%)
Neutropenia	4 (11.11%)	5 (13.89%)	2 (5.56%)	0 (0.00%)	3 (7.89%)	5 (13.16%)	3 (7.89%)	0 (0.00%)
Febrile Neutropenia	1 (2.78%)	1 (2.78%)	1 (2.78%)	0 (0.00%)	0 (0.00%)	2 (5.26%)	0 (0.00%)	0 (0.00%)
Thrombocytopenia	1 (2.78%)	0 (0.00%)	0 (0.00%)	0 (0.00%)	0 (0.00%)	0 (0.00%)	0 (0.00%)	0 (0.00%)
Nausea	11 (30.56%)	17 (47.22%)	2 (5.56%)	0 (0.00%)	12 (31.58%)	15 (39.47%)	1 (2.63%)	0 (0.00%)
Vomiting	9 (25.00%)	15 (41.67%)	0 (0.00%)	0 (0.00%)	13 (34.21%)	11 (28.95%)	0 (0.00%)	0 (0.00%)
Constipation	3 (8.33%)	1 (2.78%)	0 (0.00%)	0 (0.00%)	7 (18.42%)	2 (5.26%)	0 (0.00%)	0 (0.00%)
Diarrhea	12 (33.33%)	5 (13.89%)	1 (2.78%)	0 (0.00%)	14 (36.84%)	1 (2.63%)	0 (0.00%)	0 (0.00%)
Abdominal pain	6 (16.67%)	0 (0.00%)	0 (0.00%)	0 (0.00%)	5 (13.16%)	0 (0.00%)	0 (0.00%)	0 (0.00%)
ALT/AST increased	5 (13.89%)	1 (2.78%)	0 (0.00%)	0 (0.00%)	4 (10.53%)	1 (2.63%)	0 (0.00%)	0 (0.00%)
Acute kidney injury	1 (2.78%)	0 (0.00%)	0 (0.00%)	0 (0.00%)	2 (5.26%)	0 (0.00%)	0 (0.00%)	0 (0.00%)
Fatigue	20 (55.56%)	12 (33.33%)	3 (8.33%)	0 (0.00%)	24 (63.16%)	9 (23.68%)	3 (7.89%)	0 (0.00%)
Dizziness	6 (16.67%)	2 (5.56%)	0 (0.00%)	0 (0.00%)	4 (10.53%)	0 (0.00%)	0 (0.00%)	0 (0.00%)
Arthralgia	4 (11.11%)	0 (0.00%)	0 (0.00%)	0 (0.00%)	7 (18.42%)	1 (2.63%)	0 (0.00%)	0 (0.00%)
Myalgia	5 (13.89%)	2 (5.56%)	0 (0.00%)	0 (0.00%)	7 (18.42%)	3 (7.89%)	0 (0.00%)	0 (0.00%)
Bone pain	5 (13.89%)	1 (2.78%)	0 (0.00%)	0 (0.00%)	10 (26.32%)	1 (2.63%)	0 (0.00%)	0 (0.00%)
Sensory neuropathy	11 (30.56%)	5 (13.89%)	0 (0.00%)	0 (0.00%)	17 (44.74%)	8 (21.05%)	0 (0.00%)	0 (0.00%)
Dyspnea	4 (11.11%)	3 (8.33%)	0 (0.00%)	0 (0.00%)	4 (10.53%)	1 (2.63%)	0 (0.00%)	0 (0.00%)
Alopecia	2 (5.56%)	34 (94.44%)	0 (0.00%)	0 (0.00%)	3 (7.89%)	35 (92.11%)	0 (0.00%)	0 (0.00%)
Skin rash	5 (13.89%)	2 (5.56%)	0 (0.00%)	0 (0.00%)	3 (7.89%)	3 (7.89%)	0 (0.00%)	0 (0.00%)

*ALT/AST* Alanine aminotransferase/Aspartate aminotransferase.

### Serum metformin concentration

**Figure 3:**
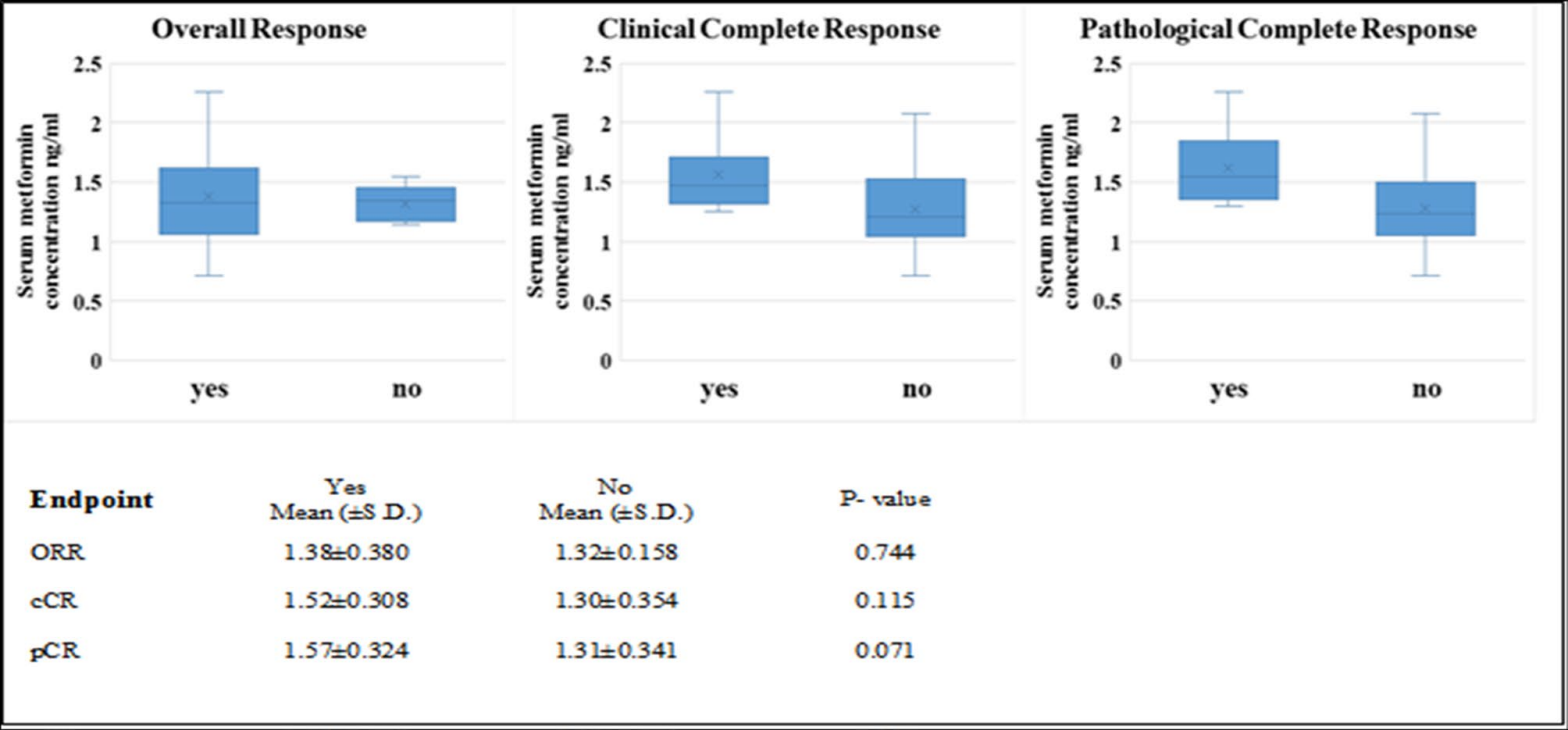
Box plot and whiskers of the relation between serum metformin level and the response of the different endpoints.

Three samples were inadequately withdrawn and were excluded from the analysis. The mean concentration was 1.36 ng/ml (± 0.35) with a range from 0.71 ng/ml to 2.08 ng/ml. Figure 3 shows the level of circulating metformin in correlation with the tumor response. Even though it was observed that the number of patients with higher circulating levels of metformin experienced better response, non-significant relation was observed between the serum concentration and the tumor response.

### Assessment of the QoL using QLQ-C30 and QLQ-BR23

Concerning the EORTC QLQ-C30, baseline mean scores for global QoL, functional scales, and symptoms scales were almost similar in the metformin group and the control group. Additionally, there was no significant difference between the mean scores of the PROs in the two groups from baseline to the last chemotherapy cycle in any of the EORTC QLQ-C30 questionnaire scales as shown in Table 5.

**Table 5:** Comparison of the mean scores of EORTC QLQ-C30 global QoL, functional, and symptoms scales between the metformin group and the control group at baseline and last chemotherapy cycle.

Variable	Baseline mean scores ± SD		Last cycle mean scores ± SD		*P*-value
	Metformin group	Control group	Metformin group	Control group	
**Global QoL and Functional Scales**					
Global Health	69.8 ± 10.9	68.3 ± 10.8	58.3 ± 12.6	57.1 ± 12.1	0.841
Physical function	76.8 ± 19.3	77.1 ± 17.2	61.6 ± 19.1	66.3 ± 18.7	0.077
Role function	85.4 ± 15.1	81.6 ± 22.9	74.5 ± 25.3	73.3 ± 27.6	0.433
Emotional function	46.1 ± 26.4	45.4 ± 22.1	52.1 ± 21.2	53.2 ± 24.3	0.941
Cognitive function	75.3 ± 19.9	73.3 ± 20.3	75.4 ± 17.2	70.0 ± 20.7	0.078
Social function	70.1 ± 26.4	74.1 ± 23.2	57.1 ± 26.9	62.1 ± 22.9	0.598
**Symptom Scales**					
Fatigue	35.2 ± 20.5	40.7 ± 25.3	61.3 ± 25.5	60.8 ± 27.3	0.442
Nausea and Vomiting	3.4 ± 14.2	5.1 ± 12.01	56.3 ± 30.2	50.4 ± 35.1	0.377
Pain	27.7 ± 25.2	31.6 ± 25.4	35.0 ± 24.9	41.2 ± 31.3	0.462
Dyspnea	12.8 ± 22.2	14.5 ± 27.1	23.3 ± 25.2	23.9 ± 27.3	0.841
Insomnia	41.8 ± 28.7	39.3 ± 19.3	43.3 ± 28.4	40.0 ± 26.3	0.646
Appetite loss	13.7 ± 25.9	19.6 ± 29.1	72.5 ± 25.9	67.5 ± 32.4	0.489
Constipation	10.3 ± 21.6	17.9 ± 30.2	18.3 ± 27.2	28.3 ± 38.9	0.602
Diarrhea	4.2 ± 13.5	3.4 ± 10.1	21.6 ± 29.7	16.7 ± 26.1	0.464
Financial difficulties	32.4 ± 26.1	27.3 ± 28.4	40.8 ± 31.5	38.3 ± 34.2	0.756

*QoL* quality of life, *EORTC QLQ* European Organization for Research and Treatment of cancer quality of life questionnaire, *SD* standard deviation, *P*-value: the variance from baseline between the two groups using ANCOVA test.

On the other side, there was no observed significant difference in the functional scales PROs of the EORTC QLQ-BR23 tool between the metformin group and the control group, considering baseline mean scores of the PROs as the covariates. Besides, the two functional scales, sexual functioning and sexual enjoyment were neither feasible for scoring nor for statistical analysis as most of the patients refused to respond to their questions. Additionally, no statistically significant difference between the two groups in the symptoms scale was observed in the breast symptoms, arm symptoms, upset by hair loss, and systematic therapy side effect as shown in Table 6.

**Table 6:** Comparison of the mean scores of EORTC QLQ-BR 23 functional and symptoms scales between the metformin group and the control group at baseline and last chemotherapy cycle.

Variable	Baseline mean scores ± SD		Last cycle mean scores ± SD		*P*-value
	Metformin group	Control group	Metformin group	Control group	
**Functional Scales**					
Body image	84.8 ± 20.4	86.2 ± 17.8	60.6 ± 15.7	64.3 ± 13.8	0.278
Future perspective	40.8 ± 33.3	44.16 ± 34.9	47.5 ± 28.1	50.0 ± 29.2	0.863
**Symptom Scales**					
Upset by hair loss	3.3 ± 10.1	6.6 ± 15.4	81.6 ± 23.8	84.1 ± 21.3	0.744
Systematic therapy side effect	11.4 ± 7.4	12.8 ± 8.1	53.2 ± 16.9	60.2 ± 17.2	0.104
Breast symptoms	21.25 ± 27.7	17.9 ± 21.5	14.1 ± 18.7	13.5 ± 16.6	0.186
Arm symptoms	24.7 ± 28.5	25.2 ± 29.9	19.4 ± 23.2	18.1 ± 23.4	0.218

*EORTC QLQ* European Organization for Research and Treatment of cancer quality of life questionnaire, *SD* standard deviation, *P*-value: the variance from baseline between the two groups using ANCOVA test.

## Discussion

Previous breast cancer (BC) researches proposed that metformin possesses both direct and indirect anticancer mechanisms. The direct anticancer effects are particularly through inhibition of AMPK/mTOR pathway^47^. On the other side, the indirect effects on cancer cells are exhibited by its ability to lower the blood glucose level, inflammatory molecules, insulin level, and circulating insulin-like growth factors (IGF)^48–50^. Additionally, metformin has shown preclinical broad effects on multiple targets of the deregulated lipid and carbohydrate metabolism associated with BC^51^.

Stratified randomization prevents imbalance between treatment groups for known factors that influence prognosis or treatment responsiveness. In the literature, different factors could influence the response of either the anticancer therapy or the anticancer effects of metformin. The maximum desirable number of strata is unknown, but experts argue for keeping it small^52^. In the current study, the stratification was performed based on the factors that could affect the response of metformin in BC patients including HER2 status, BMI, and menopausal state^53^. The ALTTO study and the study by Kim et al. (2015) showed that the survival outcomes were significantly enhanced in diabetic patients with HER2 + BC following the addition of metformin, and particularly with HR + ^3,8^. The insulin-like growth factor 1 (IGF-I) receptor, insulin receptor, and HER2 act through the same downstream signaling pathway via phosphatidylinositol 3-kinase (PI3K)/AKT/mTOR; the presence of interactions at multiple levels between PI3K/AKT/mTOR, estrogen, growth factor signaling, and the receptor tyrosine kinase cascade stimulates cancer cell proliferation and survival. As metformin can suppress one or more of these signaling pathways, it shows anticancer effects, particularly in HER2 + BC. Although BMI might be a reliable marker for potential diabetes, several studies have shown beneficial anticancer effects of metformin in obese and overweight BC patients^9,54^. Obesity is responsible for increasing the production of estrogen, and consequently, stimulating estrogen-dependent tumor growth, and adipocytes are the main source of the enzyme aromatase responsible for converting androgens to estrogens. Furthermore, the higher the adiposity, the more insulin resistance and hyperinsulinemia, which in turn increase the levels of IGF1 and BC cell proliferation^55^. Hence, metformin may exert an anticancer effect in obese patients through the reduction of insulin resistance and its antiproliferative activity. Moreover, Goodwin et al. (2008) found that insulin levels were significantly higher in postmenopausal compared to premenopausal BC patients^56^. Furthermore, the study by Yam found that obesity contributes to worse outcomes in postmenopausal women with breast cancer^9^.

In the current study, it was noted that some factors showed varying proportions between the two groups, such as histological grade and intrinsic subtype, as these factors were not included in the stratification factors. Even though their total percentage could be comparable to the literature^57,58^, it was found that HER2 +/non-luminal and TNBC were more common in the control group, which may be more likely to result in a better response to the chemotherapy rather than a difference in the efficacy of metformin.

The first clinical evidence of the anticancer properties of metformin, Jiralerspong et al. (2009) observational study showed a significant correlation between the addition of metformin to the NACT and the pCR rates in diabetic BC patients. However, in the current study, it was observed that the clinical and pathological response values were enhanced numerically but not significantly by metformin use in operable locally advanced BC cohorts receiving anthracycline/taxane-based chemotherapeutic regimen, in agreement with the METTEN study^12^. Moreover, the MYME trial failed to provide evidence to support the anticancer effects of adding metformin to the first-line chemotherapy treatment in metastatic BC^7^. This result could be due to the decrease of the potential role of metformin on host metabolism to modulate the response to chemotherapy in the presence of aggressive tumor load as in advanced BC^7^. In addition, Pimentel et al. (2019) did not recommend the use of metformin in non-diabetic metastatic BC patients, as no beneficial effect of the combination of metformin with chemotherapy was observed^59^.

Zhao et al. (2017) proposed that higher doses of metformin can exert its direct anticancer effects on AMPK–mTOR pathway^60^. Therefore, 2gm/daily dose was administered to the patients in the current study. The interpatient pharmacokinetic variability of metformin serum concentration could be experienced in cancer patients receiving the same dosage, due to abnormalities in absorption, distribution, elimination, and protein binding that could affect their response to the same dose of metformin^32^. Moreover, identifying genetic differences in drug metabolism may be particularly fruitful in understanding pharmacokinetic variability. The antitumor activity of metformin could also be dependent on the cellular uptake of the drug, which is primarily regulated by the membrane transporter organic cation transporter1 (OCT1), because of metformin hydrophilic and cationic composition^61,62^. Some clinical trials referred to the possible influence of OCT1 expression on the response of BC patients to metformin as an anticancer agent, which in some individuals or BCs may be altered to be more or less effective in transporting metformin into the cell by polymorphism or genetic error^16,63^.

In the current study, it was observed significantly higher pCR in patients with HER2 + BC irrelevant to metformin use. In addition, it was found that high baseline levels of Ki67 were associated significantly with pCR. Previous studies have shown that axillary pCR was associated with high levels of Ki67 expression, high histologic grade, ER-negativity, and HER2-positivity^64,65^. Another trial showed that increased expression of Ki67 is an index of good prognosis in patients responding to chemotherapy, but an index of poor prognosis in patients with no response to chemotherapy^66^.

Moreover, and in agreement with previous studies, it was found that high-grade tumors were significantly associated with pCR^67,68^. A high histological grade can predispose to superior chemotherapeutic response because of either their independent influence on the tumor’s sensitivity to chemotherapeutic treatment or being a predictor for triple-negative tumor cells molecular subtype that is associated with better response to chemotherapy^68^. Additionally, the Clustered Neo-Bioscore trial turned to a better understanding of the prognostic factors for women with non-pCR carcinoma which showed a good response in high-grade tumors^69^. In the current study, the increased proportion of high histological grade in the metformin group might be one of the reasons for the higher pCR in this group.

Goorts et al. (2017) indicated that the clinical tumor size is the most important prognostic factor for pCR^67^. However, the present study could not find a correlation between tumor size and pCR. This could be justified because the selected patients in this study were with proven locally advanced BC. Thus, the cases of small tumor size were associated with the high nodal stage or skin infiltration.

In agreement with Martin-Castillo et al. (2018), the BCR was higher numerically in the metformin group compared to the control group. However, this superiority in BCR should not be treated as a true clinic-molecular benefit of the metformin users. As it is known that breast conservation depends on numerous factors that include tumor size and location, the presence of ductal carcinoma in-situ, the presence of multifocal lesions, and the compliance of patients^70^.

On the other side, the most common adverse events in both groups were blood disorders, GIT side effects, neuropathic events, fatigue, and hair loss. It is worth mentioning that the metformin group experienced worse GIT symptoms in agreement with the literature^7,12,16,59,71^. Besides, it was observed a lower incidence of adverse events; arthralgia, myalgia, and bone pain in the metformin group in comparison to the control arm. These results are supported by preclinical studies, which observed the ability of metformin to prevent or treat osteoarthritis through the attenuation of osteoarthritis structural worsening and the modulation of pain^72^. This beneficial effect could be due to the activation of AMPK, which has a potential therapeutic target for osteoarthritis^72^. Besides, even though chemotherapeutics are associated with a significant loss in bone mineral density of BC patients^73^; fortunately, metformin improves the quality of bones and reduces fracture risk. A potential reason for this effect is the key role of AMPK in signaling pathways involved in bone physiology^74^. Hence, metformin can be used as an adjuvant treatment in bone disorders, and can decrease bone metastases^75^.

In addition, patients receiving metformin were experiencing fewer and lower intensity events related to neuropathy. A possible explanation for this improvement in the metformin group can be deduced from the following premises. Chemotherapy-induced peripheral neuropathy (CIPN), characterized by loss of sensory sensitivity and pain in hands and feet, is a major dose-limiting toxicity associated with many chemotherapeutics^76^. Mao-Ying et al. (2014) preclinical study concluded that metformin possesses an underlying neuroprotective beneficial mechanism, due to the reduction of peripheral nerve endings loss^76^. Also, AMPK is capable of regulating a variety of cellular processes such as mitochondrial metabolism and protein translation, in which many of them are thought to contribute to pathological pain including CIPN^77^. Given the effect of metformin on the activation of AMPK, therefore it can lead to the reduction of neuropathic events. Moreover, clinically, metformin has shown a decrease in daily pain scores of diabetic patients suffering from low-back pain^78^. Therefore, metformin is thought to have an anti-neuropathic pain effect in clinical populations. Clearly, future prospective studies are needed to understand the association between the use of metformin and neuropathic pain.

The HRQoL is a multidimensional construct covering at least several key dimensions, such as disease-related and treatment-related symptoms, physical, psychological and social functioning^79^. Therefore, one of the main objectives in the treatment of BC patients is to maintain their QoL.

Patient-reported outcome (PRO) is a type of clinical outcome assessment that examines how a patient feels or functions directly from the patient without interpretation by others, and has been acknowledged by the food and drug administration (FDA) as an important approval endpoint^80,81^. Thus, a PRO measure is an effective instrument for eliciting information from patients about treatments and their benefits in ways that healthcare practitioners are frequently unaware. The impact on QoL is a considerable point when weighing the risks and benefits of breast cancer therapy^82^. As the case with breast cancer symptoms, these adverse events interfere with the daily activities and impose considerable challenges on breast cancer patients. When presenting a cancer treatment's benefit to regulators, providers, payers, and ultimately to patients, it is ideal that the treatment improves survival and suppresses tumor development while also protecting patients' quality of life, symptom experience, and overall perceived health status. As metformin could worsen some adverse events related to GIT symptoms, therefore, PROs could affect the compliance and adherence of the patients to their treatment, as well as their concordance. Hence, a trade-off between the risk and benefit of metformin addition to a highly emetogenic chemotherapeutic regimen on the outcomes of BC patients is needed. In the current study, PROs were examined as a secondary outcome for breast cancer patients.

In the literature, the effect of metformin on the quality of life of BC patients was conducted by only two trials using the core questionnaire EORTC QLQ-C30^59,83^. Both trials observed non-significant difference in the QoL of BC patients by the addition of metformin to their treatments. However, they did not use a specific tool for BC patients in the evaluation. Therefore, it is of vital importance to evaluate the QoL of BC patients using the breast cancer specific module, EORTC QLQ-BR23 module.

Following the EORTC QLQ-C30 scales, the present study revealed no clinically or statistically significant variance from baseline between the metformin group and the control group. Functional scales showed various results between the two groups but without achieving a level of significance. In agreement with the results of Pimentel et al. (2019), it was observed a non-significant deterioration of physical function scores from baseline in the metformin group compared to the control group. In addition, it was observed a non-significant improvement in the cognitive function of BC patients receiving metformin. A potential justification by Lin et al. (2018), which concluded an improvement in the cognitive function of patients with non-dementia vascular cognitive impairment and abnormal glucose metabolism receiving metformin^84^. This could be explained by the ability of metformin to penetrate rapidly the blood–brain barrier, protect neurons via anti-inflammatory action, and improve brain energy metabolism^84^. However, we should cautiously deal with these results, as the patients’ characteristics were different from the current study such as the diabetic and the cognitive states. Additionally, Hartman et al. (2019) found that metformin might not positively affect neurocognitive functioning among BC survivors in general. However, verbal functioning could be enhanced by weight loss among individuals with higher BMI^85^.

Besides, the emotional function last cycle mean scores enhanced numerically from the baseline values in both groups of patients, irrelevant to the metformin use. That could be due to the decrease of anxiety and depression scores among the patients, as all the patients were recently informed about their ominous disease just before being enrolled in the study. Although AlHussain et al. (2020) deduced that metformin has an antidepressant effect in non-diabetic patients with polycystic ovarian syndrome, no observed significant difference was reported between the two groups of BC patients enrolled in the present study^86^. Moreover, it was noted a decrease in social functioning scores from baselines among the participants of the two groups of patients. That deterioration in social function Likert scales suffered by the patients could be related to the adverse effects of the chemotherapy administration especially hair loss and fatigue.

As well, variance from baseline between the metformin group and the control group of the symptoms scales did not reach a significant level. It is also known that metformin causes GIT disturbances and elevated homocysteine levels, which in turn can cause chronic fatigue^87^.

It was observed that insomnia symptom scales did not show a statistically significant difference between the metformin group and the control group. This result is against the Wiwanitkit et al. (2012), which showed that metformin, the insulin sensitizer, can lead to sleep disturbance, which may affect patterns of normal dreams^88^.

The strength of this trial included the homogeneity of the treatment received by the patients in the study, and the homogenous patient population since all the patients were nondiabetic with proven locally advanced BC. In addition, following the CONSORT guidelines methodology in the trial. Furthermore, the stratification criteria could give a trustworthy prediction model of the potential prognostic factors that were associated with metformin anticancer activity in BC settings in the previous trials^8,9,54^. However, the study was limited by the stratification factors that could affect the response to metformin rather than the response to chemotherapy in order to avoid over-stratification. It is noteworthy that most low- and middle-income countries in Africa experience severe limitations with drug access^89^, thus the availability of the anti-HER2 therapy for the patients in our hospital is post-surgery.

The current trial, based on the preliminary statistical setting, did not prove a significant effect in the clinical response and the pathological response in locally advanced breast cancer patients receiving neoadjuvant chemotherapy and metformin. However, it was found that the primary outcomes of the study enhanced numerically. Thus, larger clinical trials are needed to determine whether the non-significance is due to the lack of beneficial effect of metformin or due to the effectiveness of metformin did not reach the expected response of the current trial. It is also recommended to stratify the patients based on the factors that could affect the response to the anticancer therapy such as intrinsic subtypes.

The improvement of response in the metformin group was observed in some cohorts of patients including HER2 overexpression, also patients without or with low nodal involvement (N0-N1) at baseline, which could need further investigation. Furthermore, the serum metformin concentration should be correlated to the response of the patients. Additionally, more studies are needed to assess the beneficial effect of metformin on cognitive function, osteoarthritis, bone pain, and CIPN.

## Conclusion

Although metformin combination with anthracycline/taxane chemotherapy did not show significant improvement in the clinical and pathological tumor responses as well as the BCR of operable locally advanced BC, it was observed that all the outcomes were enhanced numerically in the metformin group compared to the control group.

On the other side, the main prognostic factors of locally advanced breast cancer response to anthracycline/taxane-based neoadjuvant treatment include HER2 + BC, high baseline Ki67, and higher histological grade. Moreover, the addition of metformin to a complex chemotherapy regimen was safe and tolerable. Metformin users experienced  a worsening of the GIT symptoms and an improvement in CIPN, bone pain, myalgia, and arthralgia. No significant effect was associated with metformin use on the quality of life of breast cancer patients. Finally, metformin was observed to be safe and tolerable, and could be added to the neoadjuvant chemotherapeutic regimen.
